# Collective Epithelial Migration Drives Kidney Repair after Acute Injury

**DOI:** 10.1371/journal.pone.0101304

**Published:** 2014-07-10

**Authors:** Aurélien Palmyre, Jeongeun Lee, Gennadiy Ryklin, Troy Camarata, Martin K. Selig, Anne-Laure Duchemin, Paul Nowak, M. Amin Arnaout, Iain A. Drummond, Aleksandr Vasilyev

**Affiliations:** 1 Nephrology Division, Department of Medicine, Massachusetts General Hospital, Boston, Massachusetts, United States of America; 2 Department of Pathology, Massachusetts General Hospital, Boston, Massachusetts, United States of America; 3 Department of Biomedical Sciences, NYIT COM, Old Westbury, New York, United States of America; 4 Department of Developmental and Regenerative Biology, Harvard Medical School, Boston, Massachusetts, United States of America; 5 Department of Genetics, Harvard Medical School, Boston, Massachusetts, United States of America; University of Cambridge, United Kingdom

## Abstract

Acute kidney injury (AKI) is a common and significant medical problem. Despite the kidney’s remarkable regenerative capacity, the mortality rate for the AKI patients is high. Thus, there remains a need to better understand the cellular mechanisms of nephron repair in order to develop new strategies that would enhance the intrinsic ability of kidney tissue to regenerate. Here, using a novel, laser ablation-based, zebrafish model of AKI, we show that collective migration of kidney epithelial cells is a primary early response to acute injury. We also show that cell proliferation is a late response of regenerating kidney epithelia that follows cell migration during kidney repair. We propose a computational model that predicts this temporal relationship and suggests that cell stretch is a mechanical link between migration and proliferation, and present experimental evidence in support of this hypothesis. Overall, this study advances our understanding of kidney repair mechanisms by highlighting a primary role for collective cell migration, laying a foundation for new approaches to treatment of AKI.

## Introduction

Acute kidney injury (AKI) is a very common medical problem resulting in significant morbidity and mortality [1], [2]. The current treatment of AKI is predominantly supportive [3], [4].The kidney has a remarkable ability to repair, and patients that can be successfully supported have a good chance of recovering adequate kidney function. However, despite significant efforts towards improving early diagnosis of AKI [5] to limit the severity of the illness, early detection and prevention of acute kidney injury is not always possible and the mortality rate for the AKI patients who require dialysis is still 50–80% [4]. Thus, there remains a need to develop strategies to enhance the intrinsic ability of kidney nephrons to regenerate.

Recent studies have suggested that, following an ischemic kidney injury, remaining epithelial cells repopulate the injured tubule without a contribution from stromal or circulating progenitor cells [6], [7]. Therefore, identifying the basic mechanisms governing the intrinsic epithelial restitution is central to understanding how the kidney recovers from AKI and to designing optimal strategies for treatment of patients with AKI.

It has been long acknowledged that cell proliferation plays a major role in kidney recovery from acute injury [8], [9]. Additionally, based on indirect evidence, cell migration has been suggested to be a component of kidney repair [10]. Another potential process that may play a prominent role in kidney repair is epithelial de-differentiation and metaplasia [8]–[10]. However, the relative importance of these processes in kidney repair remains unknown, in part due to the limitations of mammalian AKI models where precise spatio-temporal control and visualization of repair mechanisms remain challenging. To address the relative roles of cell migration, cell proliferation and cell metaplasia in kidney repair, we designed a novel assay of segmental acute kidney injury using the zebrafish pronephros as a model system.

The pronephric kidney in larval zebrafish is a mature functioning organ that contains segments similar to the mammalian nephron, including a glomerulus, proximal and distal tubules and a collecting duct [11]. Thus, larval pronephric kidney (5–14 dpf) can be utilized to study cellular and molecular processes involved in kidney injury and repair. The most common model to study kidney injury in zebrafish is a gentamicin model [12], [13]. It has been used successfully to screen for compounds that might enhance kidney repair process [14]. Despite being a very powerful model, it does not allow a precise spatiotemporal control of the injury. This makes it difficult to study cellular and molecular processes involved in kidney repair. To overcome this limitation, we developed a method that uses a low energy targeted violet laser light (405 nm) to induce segmental ablation of GFP-expressing pronephric nephron segments. The repair process can then be directly monitored by time-lapse microscopy in these kidney-GFP fluorescent transgenic fish. Similar to other laser ablation techniques [15], this system provides significant advantages over existing models of epithelial injury. On one hand, it allows us to study *in vivo* processes in a vertebrate organism, thus overcoming limitations of cell culture assays. On the other hand, it provides spatial and temporal control over the timing and extent of injury and allows for direct visualization of repair processes rivaling that offered by *in vitro* assays. Using this method we show that collective cell migration is the first response of kidney epithelia to injury. Our results also suggest that cell migration is a primary stimulus for subsequent cell proliferation.

## Results

### A novel model of AKI based on focused violet laser photoablation

**Figure 1:**
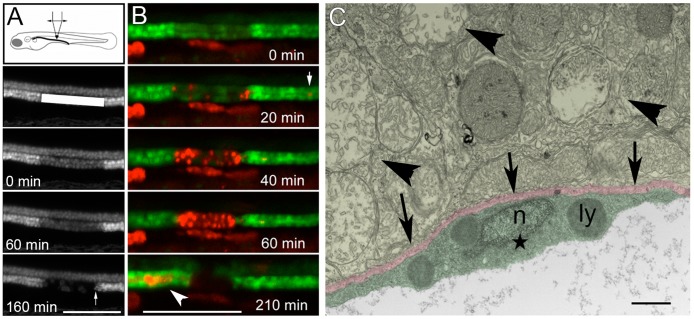
405 nm laser ablation induces kidney specific segmental epithelial injury in GFP transgenic zebrafish. (A) 12 dpf zebrafish was subjected to 405 nm laser treatment using a confocal microscope. The plane of maximal illumination was guided by kidney GFP fluorescence (here- E11-9 transgenic fish). The laser scan window is shown by the white rectangle. Efficient laser treatment was monitored by observing GFP bleaching in the ablated area (with a target of ∼50% initial reduction in GFP fluorescence). The ablated segment continued to lose GFP positivity with only an occasional cell surviving at 160 min post- laser treatment. The arrow points to a sharply defined edge of surviving epithelium. Scale bar = 70 µm. (B) Propidium iodide (PrI, red) staining confirmed that loss of GFP positivity was due to cell death, as opposed to other mechanisms. As cells in the injured segment lost GFP positivity, they became strongly PrI-positive. Every cell showed PrI positivity by 60 min post injury, and by 210 min PrI-positive material was seen exclusively in the distended lumen proximal to the obstructed segment (lower panel). PrI-positive material could also be seen transiting through the distal tubule (arrow in 20 min panel). Scale bar = 100 µm. (C) Electron microscopy of the injured epithelium 3 hours post-laser treatment shows compacted swollen degenerated mitochondria (arrowheads). The basement membrane (pink, arrows) and the adjacent, likely stromal or endothelial cell (green, star) is preserved. “n” – intact nucleus, “ly” – lysosome. Scale bar = 1000 nm.

To investigate the role of cell migration, cell proliferation and cell metaplasia in kidney repair, we developed a new *in vivo* model of segmental kidney injury using transgenic zebrafish. The transgenic zebrafish, expressing GFP in kidney tubule (ET11-9, ET33d10 and *Tg(atp1a1a.4:GFP*), were subjected to a localized violet (405 nm) laser irradiation focused on a 20–100 µm span of pronephric epithelium using a confocal microscope. This approach allowed us to target a defined group of cells (from one to hundreds) in a single window (Figure 1 A). The rationale for this method is twofold: 1) GFP fluorescence allows us to focus the laser beam with maximal intensity in the GFP-expressing tissue; 2) GFP actively absorbs light around 405 nm, presumably acting as energy sink to potentiate cell injury. The part of the tubule irradiated with the 405 nm laser can be seen just after the photoablation by observing GFP photobleaching (Figure 1 A: middle panel, B: upper panel). As we predicted, this method produced a sharply defined segment of epithelial cell death within the first 1–3 hours after irradiation (Figure 1 A,B).

To confirm cell death (as opposed to loss of fluorescence) we performed propidium iodide (PrI) staining in live zebrafish larvae. The disappearance of GFP positivity in injured cells strongly correlated with PrI staining. With a sufficient laser exposure, the entire stretch of the exposed pronephric epithelium lost GFP fluorescence and became PrI-positive within just one hour (Figure 1 B). The injured cells subsequently ‘spilled’ their content into the lumen (Figure 1 B, lower panel, arrowhead). Consistently, some cellular material (mainly the membranous component) was left behind, forming a plug that often resulted in obstructive dilatation of the epithelium upstream of the injury site (Movies S4, S5). This was similar to proteinaceous debris seen in mammalian acute kidney injury (Figure S1).

We confirmed the specificity of kidney injury by electron microscopy at 3 hours post injury (hpi). While kidney tubule cells degenerated, leaving behind compacted swollen mitochondria (Figure 1 C, arrowheads), non-epithelial cells at the injury site were preserved (Figure 1 C, star). In addition, the basement membrane was also preserved (Figure 1 C, arrows). Thus, our method allows us to induce targeted ablation of kidney epithelial cells while preserving the surrounding tissues.

### Collective cell migration during kidney repair

**Figure 2:**
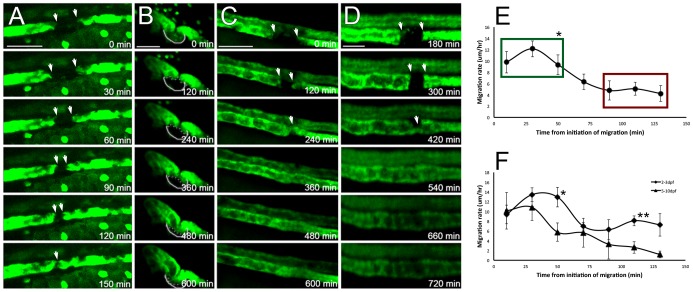
Surviving kidney epithelium responds by collective migration. Various kidney GFP transgenics were examined by time lapse confocal microscopy at different developmental stages. (A) Proximal tubule epithelium (ET33d10 transgenic, 2.5 dpf), after segmental ablation of the proximal tubule, shown in 30 min intervals. Scale bar –60 µm. (B) Proximal convoluted tubule epithelium (ET33d10 transgenic, 6 dpf), after segmental ablation of the proximal tubule, shown in 120 min intervals. Scale bar –60 µm. (C, D) Distal tubule epithelium (ET11-9 transgenic) after segmental photoablation shown in 120 min intervals in 7 dpf (C) and 5 dpf (D) fish. (D) shows marked obstructive dilatation of tubule epithelium, some of which is also seen in (C). This is a commonly observed phenomenon. Scale bars: (C) - 60 µm, (D) - 30 µm. (E) Migration rates as a function of time after the initiation of the migratory response. The rates are maximal right after the initiation of the migration and decrease over a few hours. The three early time points (green box) show statistically significant difference from the three late time points (red box, p<0.01, n = 10). The initial migration rates are similar in young (2–3 dpf) fish and older (> = 5 dpf) fish (p = 0.84), but the rates appear to persist longer in younger fish. Statistically significant differences between the rates can be seen at 50 min (p = 0.02, n = 5,5) and 110 min (p<0.01, n = 5,5) time points, as shown in (F).

We studied the behavior of the adjacent surviving epithelial cells by time lapse confocal microscopy (Figure 2, Movies S1–S5). We observed that surviving proximal and distal tubule epithelia responded by bi-directional migration to close the epithelial gap after acute injury (Figure 2 A–D). The pronephric epithelium migrated as confluent epithelial sheets, preserving cell-to-cell contacts, indicative of collective cell migration [16]. In addition, we did not see GFP-expressing cells escape confines of the tubule. The rate of the epithelial migration (average of 7.4 µm/h over the first 2 hours after the onset of migration) was similar to that observed during kidney development and *in vitro* scratch assays of kidney epithelial lines [17]. The migration started without delay immediately after the death of the injured epithelial segment. It was fastest during the first hours post-injury and slowed as migration continued (Figure 2 E,F). This pattern was different from that reported in cultured kidney epithelial cells [17], where the migration accelerated with time. We intentionally examined embryos and larvae at different times during development to determine if the migratory response was different in zebrafish of different age. The initial migration rate was independent of the age of the fish, but the migration rates declined more slowly in younger fish (2–3 dpf) compared to older ones (5–10 dpf, Figure 2 F). The peak migration rate was independent of the length of the injured segment (Figure S2). Migration continued until two surviving epithelial sheaths came in contact and re-established epithelial continuity (Figure 2, B–D). The extent of migration varied with the position of the cell relative to the migration edge. About 50 µm of the epithelium immediately adjacent to the injured segment migrated with very similar rates (Figure S3). However, further away from the migration edge, there was a significant drop in the extent of migration. As a result, the epithelial segment ∼100 µm from the migration edge appeared to experience the most migration-induced linear stretch.

### Absence of epithelial to mesenchymal transition

**Figure 3:**
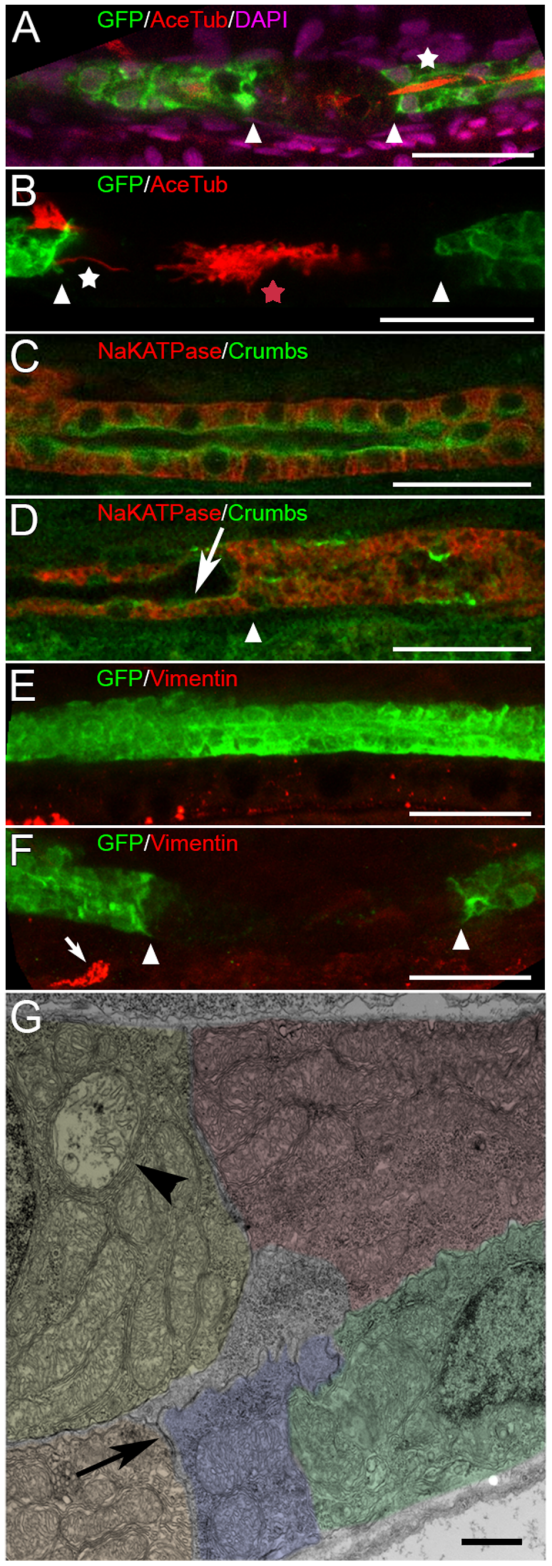
Regenerating epithelia remain differentiated. Kidney epithelium is shown at 4–6 hours post injury (hpi), on the site of the laser ablation. (A, B): Anti-GFP (green), anti-acetylated-tubulin (red) and DAPI (magenta, A) staining of the injured tubule shows that cilia and cilia bundles (multiciliated cell in A) are present at the edge (white arrowheads, also Figure S4) of surviving epithelium (white star in A and B). The aggregated cilia bundles in B (red star) are visible in the middle of the injured segment (between arrowheads). (C and D): Na/K ATPase (red) and Crumbs (green) expression in intact (C) and injured (D) epithelium (also Figure S4). AKI does not affect the expression of Crumbs on the apical surface of surviving cells (D, arrow), while its distribution is randomized in the injured segment (right of the arrowhead in D, which marks the border between the intact and the injured segment. (E, F): Vimentin is not expressed in intact kidney epithelium (E) and is not up-regulated after acute injury (F, also Figure S4). Vimentin staining can be seen outside of the kidney (arrow in (F)). Bar lengths in (A-F) are 30 µm. (G) Electron microscopy of a longitudinal section at the edge of surviving epithelium (the edge itself is not shown, to the left). Apical junctional complexes (arrow) are preserved. Bar length is 1 µm. One degenerated mitochondrion indicates partial injury to the cell at the edge of the injury (arrowhead). Different colors are used to delineate individual epithelial cells. The virtual slice thickness in (A-F): A- 1.4 µm; B-5.6 µm (7 slices); C,D- 1.4 µm; E- 7.0 µm (8 slices); F- 11.2 µm (15 slices).

It has been proposed that induction of cell migration may require loss of epithelial identity and acquisition of a more mesenchymal phenotype [9], [10], [18]. To investigate whether epithelial to mesenchymal transition occurs during kidney migration after segmental photoablation, we stained migrating epithelia for a number of epithelial and mesenchymal markers. Cilia are localized on the apical (luminal) side of the epithelial cells. We observed that migrating epithelia retained their apical cilia even at the migrating front (Figure 3 A,B, Figure S2). Interestingly, we could observe cilia bundles and clumps in the middle of the injured segment. These likely represent degenerated cellular material as part of the “proteinacious” cast (Figure 3 B). Crumbs protein is also a polarity marker expressed on the apical membrane (Figure 3 C). After segmental injury, Crumbs continued to localize to the apical membrane of migrating cells (Figure 3 D, arrow). At the same time, we did not observe vimentin expression in the intact or injured migrating epithelia (Figure 3 E,F), while vimentin positive cells were observed outside of the kidney (Figure 3 F, arrow). In addition, ultrastructural examination revealed that migrating epithelia retained their apical junctional complexes and apical-basal polarity even at the migrating front (Figure 3 G). These results suggested that collective migration of regenerating epithelia takes place without a significant epithelial to mesenchymal transformation.

### Epithelial cell proliferation after kidney injury

**Figure 4:**
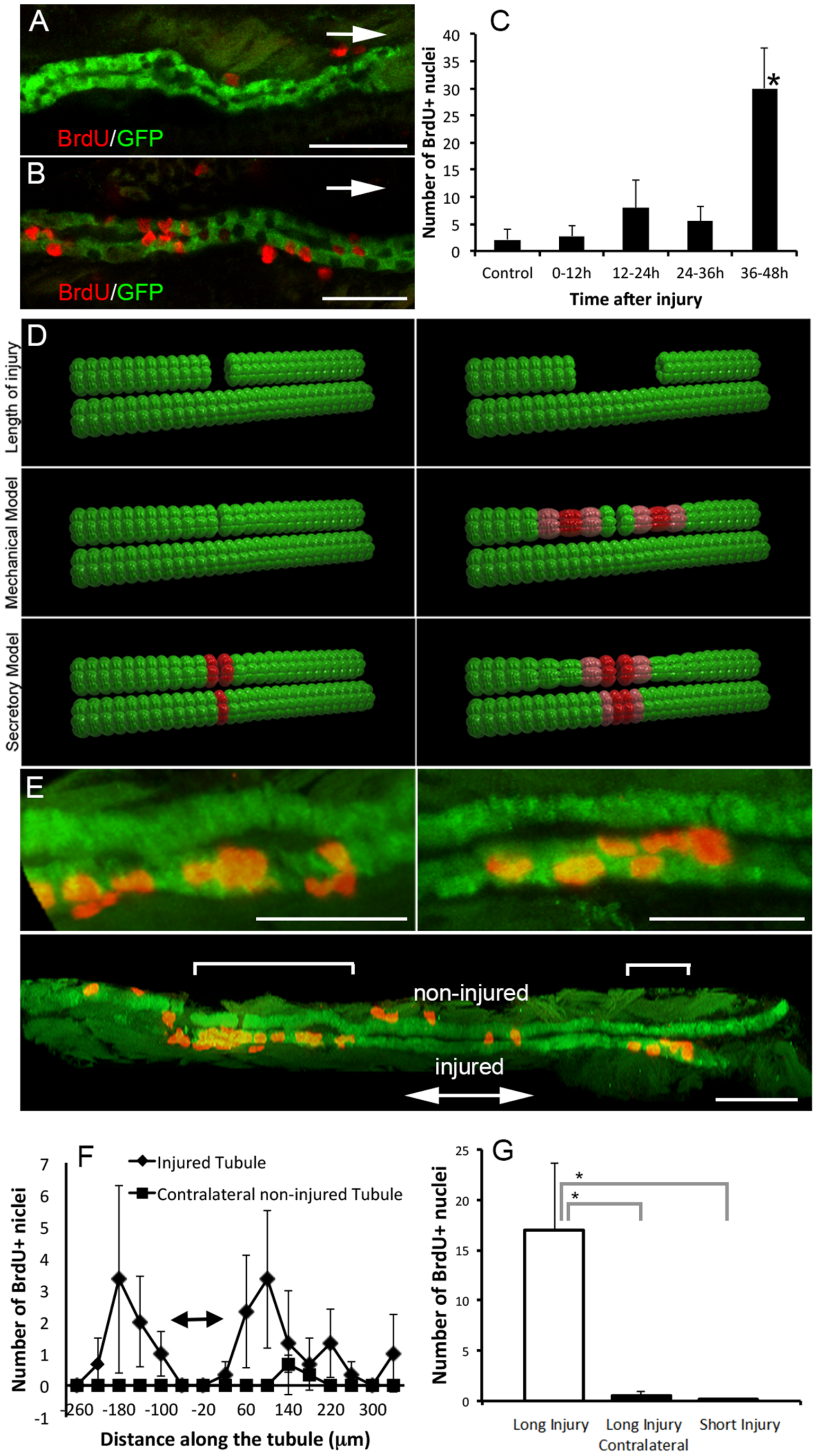
Kidney epithelial proliferation after acute injury. (A,B) Two examples of BrdU staining after segmental ablation early (0–12 hpi, A) and late (36–48 hpi, B) in ET11-9 GFP transgenic fish. Arrows indicate the direction of injury. Confocal slice thickness is 1.5 µm in (A), and 1.6 µm in (B). (C) Number of BrdU+ cells at various intervals after segmental laser ablation (0–12 hpi, n = 4, 12–24 hpi, n = 3, 24–36 hpi, n = 4, 36–48 hpi, n = 4) and compared to non-injured control condition (n = 8). There was no significant increase in cell proliferation early in the repair process (up to 36 hpi) but a pronounced increase in cell proliferation first detectable between 36 and 48 hpi, compared to control condition (p<0.05, C). (D) Two possible mechanisms that could trigger cell proliferation (mechanical stretch due to cell migration vs. a secretory factor) lead to different predictions about the distribution of cell proliferation after segmental ablation. Red color indicates the predicted distribution of cell proliferation after short (left panel) vs. long (right panel) tubule segment ablation. Upper row: initial injury; middle row: stretch response scenario (mechanical model). Lower row: secretory factor scenario. (E) Long segmental ablation resulted in two distinct bands of BrdU incorporation both upstream (left and lower sub-panels) and downstream (right and lower sub-panels, each sub-panel represents a confocal projection image). Brackets in the lower sub-panel indicate bands of proliferation upstream and downstream of the injury. The right and left upper sub-panels show a higher magnification of the areas of proliferation marked by brackets in the lower sub-panel. Other BrdU+ nuclei are outside of the kidney. (F) Comparison of BrdU incorporation in the injured (rhombi) vs. contralateral non-injured tubule (squares). This pattern of BrdU incorporation is most consistent with the mechanical model of the cell proliferation trigger. Double-arrow bar indicates the approximate site of injury. (G) Total number of BrdU+ nuclei after long (80–100 µm) vs. short (20 µm) segmental ablation (24–48 hpi). Long segmental ablation resulted in significantly increased number of BrdU+ cells compared to a contralateral non-injured side (as well as kidney epithelium after short injury or in a non-injured control). Long injury vs. contralateral non-injured tubule: p = 0.039, long injury vs. short injury: p = 0.048, n = 3 per each condition. Scale bars in (A,B and E upper sub-panels) = 30 µm, and 60 µm in (E, lower sub-panel).

To determine the temporal relationship between cell migration and cell proliferation, we examined when cell proliferation is initiated after segmental photoablation. We found that during the first 12 hours after injury, there was minimal epithelial cell proliferation in the injured tubule, similar to uninjured control larvae. In contrast, we observed marked increase in cell proliferation between 36–48 hours post injury (Figure 4 A–C). A minor increase was also observed during 12–24 and 24–36 hpi window, but it did not reach statistical significance. This result was similar to that observed in mice after ischemia-reperfusion injury [9]. Since tubule epithelial cell migration starts immediately after injury (and in most instances completes by 12 hpi) and cell proliferation does not initiate until a few hours later, we concluded that injury-induced collective cell migration occurs independently of cell proliferation.

The temporal relationship between cell migration and cell proliferation after injury was very similar to that observed in developing kidney [19]. During kidney development, cell migration stimulated cell proliferation by inducing stretch in the epithelium, secondary to cell migration [20]. It is possible that the same causal relationship exists during kidney repair. Alternatively, cell proliferation could be induced by the release of proliferation-inducing factors from injured epithelia [9]. We tested these two possible scenarios by inducing unilateral ablation in long and short segments of a tubule, while leaving the adjacent contralateral tubule intact. We reasoned that if kidney cell proliferation is controlled by mechanical stretch alone, one would expect to observe increased cell proliferation only in the injured kidney tubule, and after long but not short segment ablation, because short segment ablation would not result in significant stretching of the adjacent epithelium (Figure 4 D). In addition, increased cell proliferation would be observed both upstream and downstream of the injury site (due to bi-directional migration to close the wound). We defined a short segment ablation as 3 cell diameters, ∼20 µm, and a long segment ablation as 12–15 cell diameters, ∼80–100 µm. Based on our estimates presented in Figure S3, the short segment ablation results in <5% average linear stretch, and the long segment ablation results in ∼20% average linear stretch of the epithelium.

Our results showed that unilateral epithelial injury resulted in increased cell proliferation in the injured kidney nephron exclusively, both upstream and downstream of the injury (Figure 4 E, Movies S6, S7, S8). The average distance between the peaks of the two proliferation bands (upstream and downstream of the injury, 280 µm) corresponded to the distance between the zones of maximal stretch during migration (Figure S3, estimated: 230–300 µm). Lastly, when injury was induced in a short segment, no proliferative response was observed even in the injured kidney nephron (Figure 4 F,G). These results suggest that cell proliferation during kidney repair is primarily stimulated by mechanical factors (stretch) secondary to cell migration (and possibly also transient luminal obstruction leading to radial stretch).

**Figure 5:**
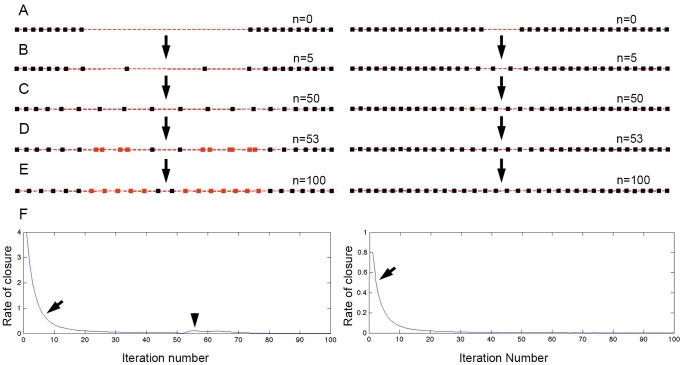
Modeling cell migration and cell proliferation during kidney repair. (A) Initial arrangement of cells in the simulation. Each square represents a single cell position. Our model is based on that published in^19^, with an addition of a ‘free edge’. (B, C) After 5 and 50 iterations of the algorithm, cells have migrated to mostly recover the epithelial gap. In the illustrated simulation, we introduced a delay in cell proliferative response to stretch. Simulations produce very similar results with or without delay (Movie S8 and S9), but presence of a delay is more reflective of the experimental observations. (D, E, left, gray markers) show divided cells in response to cell stretch after ‘long segment ablation’. (A–E, right) demonstrate that after ‘short ablation’ no cell proliferation is induced. (F) Plots of the ‘rate of migration’ after long (left panel) vs. short (right panel) segment ablation. Arrows point to a rapid decrease of the rate of migration due to cell-cell interaction. This was similar to the experimentally observed migration rate decline (Figure 2). A second small peak in migration (arrowhead) is due to cell division.

We have previously modeled the interaction between cell migration and cell proliferation during kidney development [19]. Briefly, the model presumes that cells can randomly migrate in a 2 d plane, but they restrict movement of their neighbors by imposing repulsive influence when cells get too close and attractive influence when they separate too far apart. In addition, it presumes that a low-level stochastic cell proliferative activity is signaled by spatial separation of the cells: when a cell is too distant from its neighbors, it is more likely to undergo cell division. This model can be directly applied to kidney repair by simply introducing a ‘free edge’ where no cell-cell interaction occurs (Figure 5 A, Movie S9 and S10). Indeed, the model predicted the major features of kidney repair response: slowing down of epithelial migration over time (Figure 5 F, left, Movie S9 and S10), secondary induction of cell proliferation (Figure 5 D,E, left, Movie S9 and S10), and absence of cell proliferation after short epithelial ablation (Figure 5, right panels).

## Discussion

We show here that collective cell migration is a driving force behind kidney epithelial repair and tubule resealing after acute injury. Our results suggest that cell migration is a primary, acute reparative response of injured epithelia. Collective migration in this context is likely to serve the crucial function of re-establishing a continuous barrier between the luminal tubule compartment and the interstitium. The rapidity and the robustness of this response may reflect the fact that acute kidney injury represents a biological ‘plumbing emergency’, and naked basement membrane offers no barrier to fluid and electrolyte loss. Epithelial cell migration is a robust response to wounding and injury in multiple systems [21] suggesting that this process is a highly conserved mechanism to promote regeneration. There is evidence that the mere presence of a free edge is sufficient to induce an immediate migratory response in confluent cell culture [17], suggesting that epithelia are pre-programmed to close a gap.

Interestingly, our results suggest that the reparative migratory response took place in the absence of epithelial to mesenchymal transition. This is different from what was suggested in a mouse system [9], [10], where it was proposed that cell migration might occur after transition of epithelial cells to a mesenchymal phenotype. This may be due to inter-species variation or may reflect a difference in the mode of injury in different model systems. For example, ischemia, but not cell injury per se may be the primary stimulus for epithelial to mesenchymal transition in the mouse kidney ischemia-reperfusion model [22].

It has been suggested in other systems that a purse string is a common mechanism by which epithelial closure takes place during both development [23] and repair [24], [25]. Our system excludes this possibility due to the geometry of the tubule. It is quite possible that purse string mechanism is present during small focal repair events, but the repair of large segmental tubular lesions has to take place through collective migration rather than purse string closure. Moreover, in the context of tubule regeneration after segmental injury and reestablishing nephron fluid flow, purse string closure could represent a pathologic response, because it would result in two sealed, discontinuous segments and a resultant cystic change. Similarly, luminal obstruction due to cell debris may also lead to cystic change after acute injury [20], [26], [27]. It is evident from our studies that cell debris generated by epithelial injury commonly form a ‘plug’, preventing fluid from escaping the tubule and resulting in tubule dilatation (Figure 2 C,D). This cystic change is usually transient in nature (Movie S5), but one could envision that if the mechanism of clearing the luminal debris was inhibited, this transient cystic change could lead to permanent cyst formation as can be seen in polycystic kidney disease [27]. This possibility remains to be tested in available models of PKD.

A unique feature of the injury model we present is that tubule cell death can be spatially distinguished from the initial responses of neighboring healthy cells. We observed that epithelial proliferation of cells adjacent to the wound correlated with the extent of injury and occurred in two bands around the wound site. The position of the two bands of proliferation was within the region of the epithelium experiencing the most linear stretch secondary to cell migration (Figure 4 F, Figure S3). At the same time, we observed that injuring one tubule did not result in increased proliferation in the adjacent contralateral tubule. These results suggest that the signals regulating proliferation in response to injury are intrinsic to the injured tubule itself and support the idea that proliferation may be signaled by mechanical stretch produced by cell migration. The spatial and temporal resolution of our model also allows us to propose that collective cell migration is the primary response to tubule injury, occurring well before any significant cell proliferation can be detected (Figure 4, C). Further experiments measuring cell migration and the role of mechanical stretch directly *in vivo* or in *in vitro* cell culture models will be required to definitively link cell migration and cell proliferation during kidney repair and to identify the exact mechanical forces leading to increased cell proliferation.

Another advantage of the photoablation methodology we present is that it allows graded amounts of photodamage to be applied, ranging from minimal, with no effect on cell survival to massive, resulting in cell necrosis. This gradual control is not possible using conventional photoablation techniques [15]. In addition, the method takes advantage of tissue specific GFP expression and can be potentially applied to a very large number of GFP transgenic animals already available. The Killer red fluorescent protein based system has been shown to be similarly effective for cell ablation, but the number of available fish lines is relatively small compared to a number of GFP transgenics [28]. In addition, the GFP- based system can be effectively used for both photoablation (using 405 nm laser) and subsequent imaging (using 488 nm excitation). This method may also be applicable in tissues outside the kidney. In a pilot experiment using 405 nm irradiation of heart cells expressing GFP under the control of the *cmlc* promoter, we were able to induce AV block by targeting cells around the AV canal (data not shown). This result suggests that photosensitized cell death using GFP expression may have potential for *in vivo* cell ablation in kidney and some other organs.

## Materials and Methods

This study was carried out in accordance with the recommendations in the Guide for the Care and Use of Laboratory Animals of the National Institutes of Health. The protocol was approved by the NYIT College of Osteopathic Medicine Institutional Animal Care and Use Committee (NYITCOM IACUC). All surgery and *in vivo* experimentation was performed under Tricaine anesthesia, and all efforts were made to minimize suffering.

### Zebrafish transgenic lines

The *Tg(atp1a1a.4:GFP)* transgenic line was generated as described in [29]; the ET(krt8:EGFP)sqet11–9 line and the ET(krt8:EGFP)sqet33-d10 line were a gift from Dr. Vladimir Korzh [30], [31]. All the fish lines were raised and maintained as described in [20], [32], [33]. The ET(krt8:EGFP)sqet11-9 and ET(krt8:EGFP)sqet33-d10 lines are referred to as ET11–9 and ET33d10, respectively. The *Tg(atp1a1a.4:GFP)* line is referred to as 8 kb:GFP. Embryos for the described experiments were obtained by in-crossing the heterozygous transgenic/mutant fish and selected based on presence of kidney GFP fluorescence using fluorescent dissecting microscope. Embryos were kept at 28.5°C in E3 solution during 24 h after fertilization, then media was replaced with E3 solution containing 0.003% PTU (1-phenyl-2-thiourea) to prevent pigmentation.

### Kidney injury experiments

Zebrafish were embedded in 2% low melting point agarose with 0.2 mg/ml Tricaine as previously described [33]. Pronephroi of ET11-9 transgenic zebrafish were segmentally photoablated on Zeiss LSM5 or Nikon C2 confocal microscope, using 40x water dipping objective and maximum intensity of a 405 nm laser. The 25 µs/pixel dwell time was used (Zeiss), repeated 8 times per pass (line averaging) in 2 passes. This dose of light resulted in ∼50% initial reduction in GFP fluorescence and resulted in death of exposed kidney epithelial cells. A 20–100 µm length of kidney tubule was injured in most experiments. All parameters were optimized to minimize incidental and non-specific injury.

### Immunostaining

Zebrafish were selected 3–6 hours after kidney injury. Then, they were fixed in Dent’s solution (20% DMSO, 80% Methanol) for 24 h at 4°C. They were rehydrated in 75∶25 MeOH/PBSDT, 50∶50, 25∶75 and 0∶100, followed by overnight blocking at 4°C in 10% Goat serum (Sigma) in PBSDT: 1%DMSO, 0.05% Tween20 in 1xPBS. We used the following primary antibodies: anti BrdU (Sigma), anti-GFP (Sigma), anti-Acetilated Tubulin (Sigma), anti-Crumbs (from Dr. Jarema Malicki [34]), anti-Na/K ATPase (DSHB), anti-Vimentin (Sigma). Anti-mouse and anti-rabbit secondary antibodies were used (Alexa 488 or Alexa 546 labeled, Molecular Probes). All antibody incubations were performed in 2% Goat Serum/PBSDT at 4°C overnight. All washes were done at room temperature in 2% Goat Serum/PBSDT. Imaging of antibody labeling was performed using Zeiss LSM5 or Nikon C2 confocal microscope.

### Proliferation staining (BrdU)

Zebrafish kidneys were injured at 10 dpf and incubated in 20 mM BrdU for 12–24 h, directly after injury, 12 hours post injury (hpi), 24 hpi, and 36 hpi. Zebrafish were fixed in Dent’s fixative overnight at 4°C, rehydrated in PBSDT and treated with 10 µg/ml proteinase K for 1 h30 min. Embryos were washed again and treated with 2 N HCL for 1 h. The antibody staining was performed as described above.

### Morphometry

Flattened confocal stacks were used to measure rates of epithelial cell migration by tracking a migrating edge during kidney repair. Alternatively, individual cells, symmetrically positioned around the center of injury, were traced and their inter-cell distance was determined in the beginning and at the end of migration (the time point of epithelial closure). Image analysis was performed using ImajeJ (NIH) and the results were analyzed in Excel (Microsoft). Confocal stack stitching was performed in ImageJ as described in [35].

### Transmission electron microscopy

9 days old zebrafish were injured and fixed at 3–4 hpi (overnight at 4°C) using electron microscopy fixative (2.5% glutaraldehyde, 2.0% paraformaldehyde. 0.025% calcium chloride in a 0.1 M sodium cacodylate buffer, pH 7.4). They were processed in a Leica Lynx automatic tissue processor. The larvae were post fixed with osmium tetroxide, en bloc stained with 2.0% uranyl acetate dehydrated in a graded ethanol series, embedded in pure epoxy resin and polymerized overnight at 60°C. Thin sections were cut using a diamond knife and an LKB 2088 ultramicrotome and placed on copper grids. Sections were stained with lead citrate and examined in a FEI Morgagni transmission electron microscope. Images were captured with an Advanced Microscopy Techniques 2 K digital CCD camera. Global contrast was corrected in Photoshop (Adobe Systems Inc.).

### Modeling AKI

The modeling of cell migration and proliferation during kidney regeneration was performed using Matlab software (Mathworks, Inc.). The model used was a modification of the previously published method [19] by introducing a ‘free edge’. The simulation results were exported into individual frames and reassembled into movies using ImageJ (NIH). 3 D illustrations were drafted using AOI software (http://www.artofillusion.org/).

### Statistical analysis

Statistical comparisons across experimental conditions, as well as comparisons between experimental and control conditions, were conducted using a two-tailed t-test, two-sample with non-equal variance. When comparing the experimental and control data obtained from the same fish (injured vs. non-injured tubule), we used a two-tailed paired t-test. The tests were run using Excel (Microsoft). Three to ten biological replicas were used per condition.

## Supporting Information

Figure S1 **Mouse model of AKI.** Ischemia-reperfusion results in various degrees of kidney injury. Here, severe epithelial injury is manifested by complete denudation of tubular basement membrane and formation of proteinaceous and cellular casts. Peritubular capillaries show prominent leukocyte margination. (A) – H&E, (B)-PAS stains.
(TIF)

Figure S2 **Peak migration rate as a function of the length of ablation.** Representative time-lapse confocal stacks were analyzed to determine the peak migration rate, which was plotted as a function of the ablated segment length. Linear regression equation: Y = A*X+B, A = −0.006(1/hr), B = 17.3(µm/hr).
(TIF)

Figure S3 **Extent of migration vs. distance between migrating cells.** Cells in three representative time-lapse confocal stacks were traced to determine the extent of migration as a function of the distance from the center of the injury. The initial injury length was 50–60 µm. Pairs of cells symmetrically positioned around the middle of the injured segment were traced until the epithelial gap was closed due to cell migration. The final distance between the two cells in a pair was subtracted from the initial distance and plotted on the vertical axis vs. the initial distance between the cells (gray circles). The data was then grouped based on the initial distance (50–100 µm, 100–150 µm, 150–200 µm, >200 µm), and the averages were plotted as black squares. There was no statistically significant difference between 50–100 µm, 100–150 µm and 150–200 µm groups, but the >200 µm group was significantly different from the 50–100 µm group (p<0.05). The data were fitted using linear regression, with the regression crossing the horizontal axis at 434 µm. This value provides a rough estimate of a total length of kidney epithelium that is expected to experience linear stretch due to migration. Based on this estimate, the average linear stretch due to migration is <5% (20 µm/434 µm) of the initial length in the case of a short segment ablation, and ∼20% ((80∼100 µm)/434 µm) in the case of a long segment ablation. It should be noted that a maximal linear stretch is likely larger due to uneven distribution of the cell stretch along the length of the regenerating epithelium.
(TIF)

Figure S4 **Epithelial and mesenchymal markers in injured epithelium.** (A, B) Apical cilia at the edge of surviving epithelium. Upper panel – acetylated tubulin, middle panel – GFP, lower panel – combined. Panel (B) corresponds to figure 3B. (C) Higher magnification images corresponding to figure 3D. Upper panel – acetylated tubulin, middle panel – GFP, lower panel – combined. (D) Higher magnification images corresponding to figure 3F. Upper panel – acetylated tubulin, middle panel – GFP, lower panel – combined.
(TIF)

Movie S1 **Collective epithelial migration after segmental kidney ablation.** 12 dpf ET11-9:GFP zebrafish was treated with 405 nm laser (figure 1, A) and imaged using time lapse confocal microscopy. Collective migration is present after segmental ablation. Each frame is a flattened confocal stack. Frame interval = 20 min. Number of frames = 39.
(MOV)

Movie S2 **Collective epithelial migration after segmental kidney ablation.** 2.5 dpf ET33d10:GFP zebrafish was treated with 405 nm laser and imaged using time lapse confocal microscopy. Collective migration is present after segmental ablation. Each frame is a flattened confocal stack. Frame interval = 10 min. Number of frames = 17.
(MOV)

Movie S3 **Collective epithelial migration after segmental kidney ablation.** 6 dpf ET33d10:GFP zebrafish was treated with 405 nm laser in the region of proximal convolution and imaged using time lapse confocal microscopy. Collective migration is present after segmental ablation. Each frame is a flattened confocal stack. Frame interval = 12 min. Number of frames = 66.
(MOV)

Movie S4 **Collective epithelial migration after segmental kidney ablation.** 7 dpf 8 kb:GFP-derived zebrafish was treated with 405 nm laser in the region of straight proximal-distal tubule, and imaged using time lapse confocal microscopy. Collective migration is present after segmental ablation. Each frame is a flattened confocal stack. Frame interval = 12 min. Number of frames = 60.
(MOV)

Movie S5 **Collective epithelial migration after segmental kidney ablation.** 5 dpf E11-9 zebrafish was treated with 405 nm laser and imaged using time lapse confocal microscopy. Collective migration gets initiated immediately after a drop out of the injured segment and continues until epithelial continuity is reestablished. Epithelium also shows obstructive dilatation upstream of the injury that progresses from proximal to distal as the debris move down the tubule until tubule diameter returns back to normal at the end of the recording, presumably due to clearing of the lumen. Each frame is a flattened confocal stack. Frame interval = 20 min. Number of frames = 36.
(MOV)

Movie S6 **3** **d reconstruction of E11-9 kidney tubule upstream of the prior injury site.** The zebrafish was incubated in BrdU 24–48 h post-injury and stained with anti-BrdU (red) and anti-GFP (green) antibody. All the kidney BrdU positivity is in the injured branch.
(MOV)

Movie S7 **3** **d reconstruction of E11-9 kidney tubule downstream of the prior injury site.** The zebrafish was incubated in BrdU 24–48 h post-injury and stained with anti-BrdU (red) and anti-GFP (green) antibody. All the kidney BrdU positivity is in the injured branch.
(MOV)

Movie S8 **3** **d reconstruction of E11-9 kidney tubule around the prior injury site.** The zebrafish was incubated in BrdU 24–48 h post-injury and stained with anti-BrdU (red) and anti-GFP (green) antibody. All but one kidney BrdU positive nuclei were found in the injured branch.
(MP4)

Movie S9 **Simulation of collective migration after segmental ablation.** The presence of free edge is sufficient to induce collective migration. Cell proliferation is induced after a delay due to increased cell-cell distance (stretching).
(MOV)

Movie S10 **Simulation of collective migration after segmental ablation.** The presence of free edge is sufficient to induce collective migration. In this simulation there.
(MOV)
