# Supplementary figures and images for: Collective Epithelial Migration Drives Kidney Repair after Acute Injury
Source: PLoS One. 2014 Jul 10;9(7):e101304. doi: 10.1371/journal.pone.0101304 (PMC4092191; doi:10.1371/journal.pone.0101304)

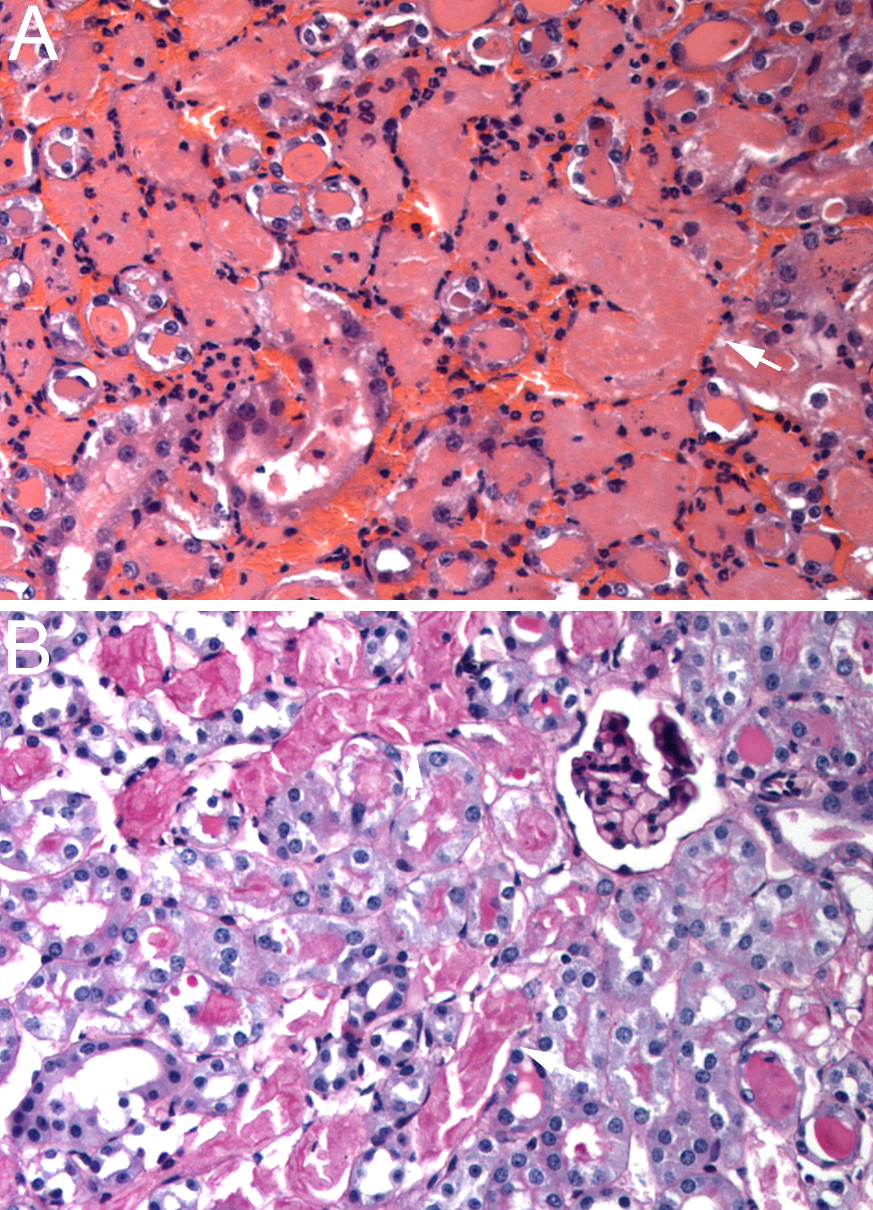

Supplement: Figure S1 — Mouse model of AKI. Ischemia-reperfusion results in various degrees of kidney injury. Here, severe epithelial injury is manifested by complete denudation of tubular basement membrane and formation of proteinaceous and cellular casts. Peritubular capillaries show prominent leukocyte margination. (A) – H&E, (B)-PAS stains. (TIF) [file pone.0101304.s001.tif]

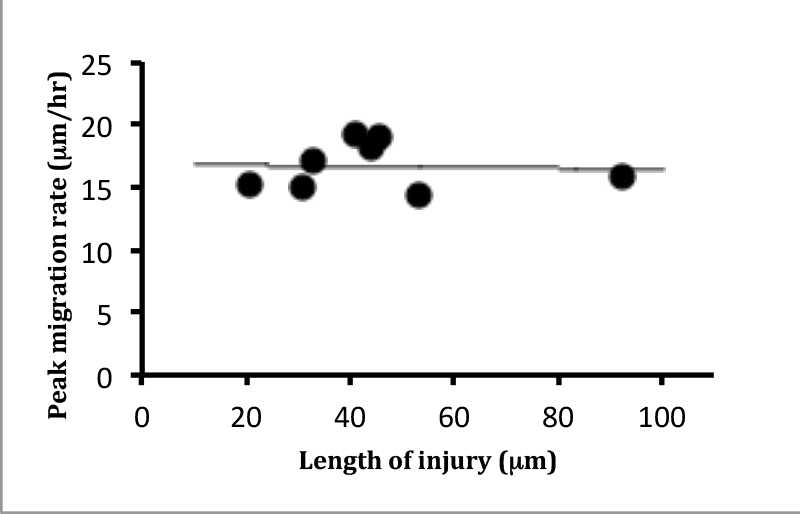

Supplement: Figure S2 — Peak migration rate as a function of the length of ablation. Representative time-lapse confocal stacks were analyzed to determine the peak migration rate, which was plotted as a function of the ablated segment length. Linear regression equation: Y = A*X+B, A = −0.006(1/hr), B = 17.3(µm/hr). (TIF) [file pone.0101304.s002.tif]

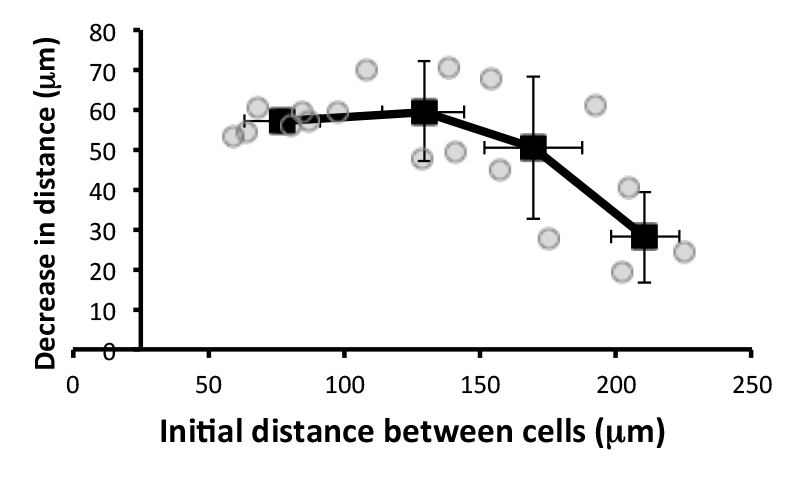

Supplement: Figure S3 — Extent of migration vs. distance between migrating cells. Cells in three representative time-lapse confocal stacks were traced to determine the extent of migration as a function of the distance from the center of the injury. The initial injury length was 50–60 µm. Pairs of cells symmetrically positioned around the middle of the injured segment were traced until the epithelial gap was closed due to cell migration. The final distance between the two cells in a pair was subtracted from the initial distance and plotted on the vertical axis vs. the initial distance between the cells (gray circles). The data was then grouped based on the initial distance (50–100 µm, 100–150 µm, 150–200 µm, >200 µm), and the averages were plotted as black squares. There was no statistically significant difference between 50–100 µm, 100–150 µm and 150–200 µm groups, but the >200 µm group was significantly different from the 50–100 µm group (p<0.05). The data were fitted using linear regression, with the regression crossing the horizontal axis at 434 µm. This value provides a rough estimate of a total length of kidney epithelium that is expected to experience linear stretch due to migration. Based on this estimate, the average linear stretch due to migration is <5% (20 µm/434 µm) of the initial length in the case of a short segment ablation, and ∼20% ((80∼100 µm)/434 µm) in the case of a long segment ablation. It should be noted that a maximal linear stretch is likely larger due to uneven distribution of the cell stretch along the length of the regenerating epithelium. (TIF) [file pone.0101304.s003.tif]

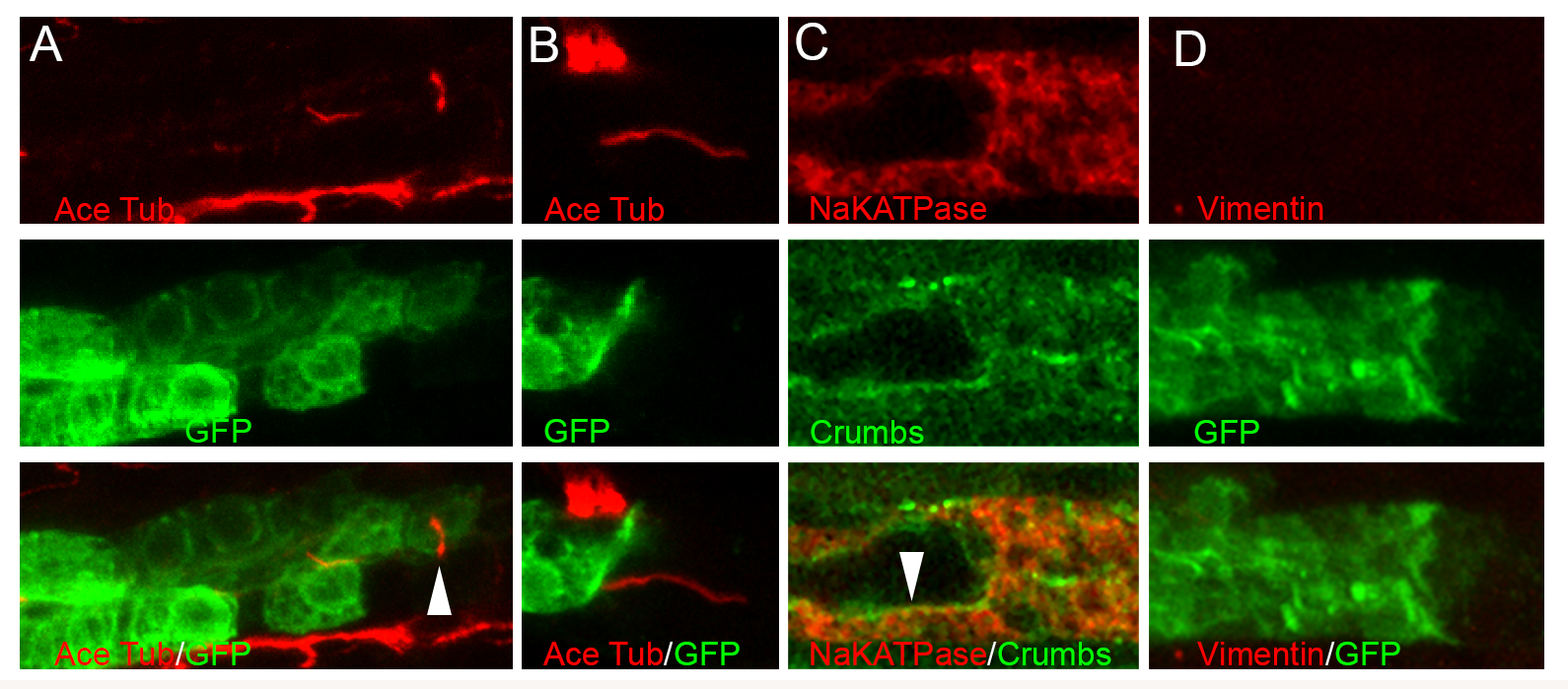

Supplement: Figure S4 — Epithelial and mesenchymal markers in injured epithelium. (A, B) Apical cilia at the edge of surviving epithelium. Upper panel – acetylated tubulin, middle panel – GFP, lower panel – combined. Panel (B) corresponds to figure 3B. (C) Higher magnification images corresponding to figure 3D. Upper panel – acetylated tubulin, middle panel – GFP, lower panel – combined. (D) Higher magnification images corresponding to figure 3F. Upper panel – acetylated tubulin, middle panel – GFP, lower panel – combined. (TIF) [file pone.0101304.s004.tif]
